# Rainbow trout (*Oncorhynchus mykiss*) muscle satellite cells are targets of salmonid alphavirus infection

**DOI:** 10.1186/s13567-015-0301-1

**Published:** 2016-01-08

**Authors:** Stéphane Biacchesi, Grégory Jouvion, Emilie Mérour, Abdelhak Boukadiri, Marion Desdouits, Simona Ozden, Michel Huerre, Pierre-Emmanuel Ceccaldi, Michel Brémont

**Affiliations:** INRA, Unité de Virologie et d’Immunologie Moléculaires, Jouy-en-Josas, France; Institut Pasteur, Unité Histopathologie Humaine et Modèles Animaux, Paris, France; UMR INRA, Génétique Animale et Biologie Intégrative, Equipe Génétique Immunité et Santé, Jouy-en-Josas, France; Institut Pasteur, Unité Épidémiologie et Physiopathologie des Virus Oncogènes, Paris, France; CNRS UMR 3569, Paris, France; Université Paris Diderot, Sorbonne Paris Cité, Cellule Pasteur, Paris, France; Institut Pasteur, Unité Recherche et Expertise Histotechnologie et Pathologie, Paris, France

## Abstract

Sleeping disease in rainbow trout is characterized by an abnormal swimming behaviour of the fish which stay on their side at the bottom of the tanks. This sign is due to extensive necrosis and atrophy of red skeletal muscle induced by the sleeping disease virus (SDV), also called salmonid alphavirus 2. Infections of humans with arthritogenic alphaviruses, such as Chikungunya virus (CHIKV), are global causes of debilitating musculoskeletal diseases. The mechanisms by which the virus causes these pathologies are poorly understood due to the restrictive availability of animal models capable of reproducing the full spectrum of the disease. Nevertheless, it has been shown that CHIKV exhibits a particular tropism for muscle stem cells also known as satellite cells. Thus, SDV and its host constitute a relevant model to study in details the virus-induced muscle atrophy, the pathophysiological consequences of the infection of a particular cell-type in the skeletal muscle, and the regeneration of the muscle tissue in survivors together with the possible virus persistence. To study a putative SDV tropism for that particular cell type, we established an in vivo and ex vivo rainbow trout model of SDV-induced atrophy of the skeletal muscle. This experimental model allows reproducing the full panel of clinical signs observed during a natural infection since the transmission of the virus is arthropod-borne independent. The virus tropism in the muscle tissue was studied by immunohistochemistry together with the kinetics of the muscle atrophy, and the muscle regeneration post-infection was observed. In parallel, an ex vivo model of SDV infection of rainbow trout satellite cells was developed and virus replication and persistence in that particular cell type was followed up to 73 days post-infection. These results constitute the first observation of a specific SDV tropism for the muscle satellite cells.

## Introduction

Sleeping disease in salmonids has been first observed in France in [1]. In rainbow trout (*Oncorhynchus mykiss*), the disease is characterized by an abnormal swimming behaviour of the fish which stay on their side at the bottom of the tanks, hence the name “sleeping” disease. This sign is presumably due to extensive necrosis of red skeletal muscle. Reported mortality rates are very variable, from negligible to over 22% in affected farms [2, 3]. The experimental infection of juvenile trout by bath immersion leads to 80% of mortality 40 days post-infection [4]. A viral aetiology of this disease has been established more than 20 years ago [5] and the virus was characterized as the first Alphavirus (*Togaviridae* family) isolated from diseased trout [6]. A genetically-related virus, the salmon pancreas disease virus (SPDV), was also described in salmon [7]. These viruses are now classified as *salmonid alphavirus* (SAV) with at least six main subtypes (SAV subtypes 1–6) where SAV1 is SPDV and SAV2 is SDV [8, 9]. Except the genome nature and its gene organization, these viruses are phylogenetically very distant from mammalian alphaviruses: larger protein size, shorter non-coding region and as a main feature they do not need any arthropod vector to be transmitted as clearly established under controlled conditions in experimental fish facilities. Different diagnostic tools have been generated allowing either the detection of the viral RNAs [10], or the viral antigens [11]. A reverse genetics system has been established for SDV allowing the manipulation of the viral genome and the expression of a reporter gene [4, 12]. The experimental transmission of the disease to juvenile trout by bath immersion is well established and reproduces each lesion type observed in the field. The histopathology in SDV infected trout is sequential (for review, [13]). Pancreatic lesions appeared first after infection followed by heart muscle lesions and finally extensive lesions of skeletal muscle fibers. Skeletal muscle lesions are characterized by degeneration and disappearance of fibers of the lateral line (red muscle) and adjacent white muscle with inflammation and fibrosis of supporting muscle fascia [14].

Immunohistochemistry analyses on organ sections from infected fish showed that viral antigens were found in the cytoplasm of the exocrine pancreas cells between 7 and 21 days post-infection and in the sarcoplasm of white and red muscle fibers between 21 and 42 days post-infection [11]. However, the cell tropism of SDV in the skeletal muscle is still unknown.

Alphaviruses affecting humans can be divided into two geographically isolated groups: New World and Old World alphaviruses. Many of the New World alphaviruses cause encephalitis, whereas the Old World viruses more typically cause fever, rash, headache, arthritis, myositis, myalgia and arthralgia [15]. Infection of humans with arthritogenic alphaviruses, such as Chikungunya virus (CHIKV), Ross River virus, O’nyong-nyong virus, Sindbis virus, and others, is a global cause of debilitating musculoskeletal diseases [15, 16]. These viruses are also of serious concern due to their ability to cause explosive epidemics that can involve millions of patients and potentially lead to emergence in new geographic regions as happened in the Indian Ocean region and more recently in America [17]. In 2005–2006, in the Reunion Island, almost 300 000 persons were infected by the CHIKV, a virus transmitted by a mosquito, *Aedes albopictus* [18]. This virus is responsible for an acute infection of abrupt onset, characterized by high fever, persistent arthritis, arthralgia, myalgia, headache, chills, photophobia and rash [19, 20]. The mechanisms by which the virus causes these pathologies are not well understood [16, 21]. Nevertheless, recent studies have provided new insights into CHIK virus pathogenesis [22]. Indeed, the diagnosis performed on muscle biopsies from infected patients with a myositis revealed two different lesion patterns: (1) atrophy and necrosis of scattered muscle fibers; (2) presence of extensive interstitial mixed acute and chronic inflammation. Immunohistochemistry analyses on the biopsies showed that viral antigens could be found inside skeletal muscle progenitor cells, designated as satellite cells, and not in muscle fibers. CHIKV also exhibits a particular tropism in vitro for satellite cells with a strong cytopathic effect, whereas myotubes are essentially refractory to infection. Such an infection of satellite cells by CHIKV could also be found in murine experimental models of infection [23]. Muscle satellite cells are myogenic precursor cells that persist in mature skeletal muscle as quiescent cells [24, 25]. They are considered as the main, if not the unique, cell type responsible for postnatal muscle growth and repair. Because CHIKV-infected cells have been observed 3 or 4 months after the acute crisis, it can be hypothesized that infection of precursor cells may have pathological consequences on long-term muscle physiology in patients. Consistent with this, susceptibility of satellite cells might be crucial for the pathophysiology of CHIKV infection in humans with a possible persistence of virus in muscle tissue leading to recurrent myalgia.

Thus, the salmonid alphavirus (SDV) and its host (rainbow trout) could constitute a relevant model to study in details the virus-induced atrophy, the pathophysiological consequences of the infection of a particular cell-type in the skeletal muscle, and the regeneration of the muscle tissue in survivors together with the possible virus persistence. We established an in vivo and ex vivo rainbow trout model of SDV-induced atrophy of the skeletal muscle. This experimental model allows reproducing the full panel of clinical signs observed during a natural infection since the virus transmission does not require any vector or virus injection, and gives the opportunity to study muscle regeneration after viral injuries. Therefore, the virus tropism in the muscle tissue was studied together with the kinetics of the muscle atrophy, and the muscle regeneration post-infection was observed. In parallel, an ex vivo model of SDV infection of rainbow trout satellite cells was developed and the virus persistence was followed up to 73 days post-infection.

## Materials and methods

### Virus and cell culture

The SDV French strain S49P used in this study has been described previously [26]. The virus was propagated in monolayer cultures of bluegill fry BF-2 cells maintained at 10 °C in Glasgow’s modified Eagle’s medium-HEPES 25 mM medium (GMEM-HEPES; Eurobio) supplemented with 2 mM l-glutamine (Life Technologies) and without foetal bovine serum (FBS; Lonza). At 7–10 days post-infection, supernatants of infected cells were harvested and virus titres were determined by plaque assays followed by an indirect immunofluorescence using the 17H23 monoclonal antibody (anti-E2 mAb) 7 days post-infection, as previously described [4, 11].

### Ethics statement

This study was performed in strict accordance with the European guidelines and recommendations on animal experimentation and welfare. All animal experiment procedures were approved by the local ethics committee on animal experimentation: COMETHEA under permit number No 12/111.

### Experimental fish infection and virus isolation

Fifty virus-free juvenile INRA synthetic strain of rainbow trout (mean weight of 0.9 or 3.3 g in SDV#1 and SDV#2 experiments, respectively) were infected by immersion in tanks filled with 3 L of freshwater with SDV (final titre, 5 × 10^4^ PFU/mL) for 2 h at 10 °C. Tanks were then filled up to 30 L with freshwater. Controls were fish mock infected with cell culture medium under the same conditions. Mortalities were recorded each day over a 60 days period. Prior to all experimental procedures, fish were anaesthetized or euthanized using 2-phenoxyethanol (diluted 1:2000 or 1:1000, respectively).

Virus isolation was performed for both experiments from kidney, spleen, heart, pancreas and ascite fluids or from serum samples for SDV#1 and SDV#2, respectively. Organs from four randomly selected individuals were removed aseptically, pooled and homogenized in a mortar with a pestle and sand in nine volumes of GMEM-HEPES (w/v) containing penicillin (100 IU/mL; Biovalley), streptomycin (100 µg/mL; Biovalley) and amphotericin B (2.5 µg/mL; Sigma-Aldrich). The supernatants were then clarified by centrifugation at 2000 × *g* for 15 min at 4 °C. Pooled serum samples were prepared from whole blood of four individuals and ascite fluids were collected from four fish. Briefly, whole blood was collected and allowed to clot and separate overnight at 4 °C and after centrifugation at 1200 ×* g* for 15 min at 4 °C, the serum was recovered. Finally, BF-2 cells were inoculated with the serum, ascite fluid or supernatant from homogenized organs diluted from serial tenfold dilutions and incubated at 10 °C for 7 days. Virus titre was then determined by immunofluorescence assay (see above).

### Fish sampling and histopathological and immunohistochemical analysis

**Figure 1:**
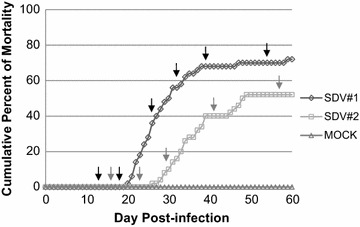
**Cumulative percent of mortality induced in rainbow trout by a natural route of SDV infection.** Juvenile rainbow trout (50 fish/tank, mean weight = 0.9 g and 3.3 g for SDV#1 and SDV#2, respectively) were infected by bath immersion with 5 × 10^4^ PFU/mL of SDV or mock-infected as a negative control. Mortalities were recorded each day over a period of 2 months and expressed as a percentage of cumulative mortality. Each infection was done in duplicate and in one of the aquariums, four fish were sampled at different time post-infection as indicated by the arrows (black for SDV#1 and grey for SDV#2).

Four infected and uninfected control fish were randomly sampled at different time post-infection: days 13, 18, 26, 32, 39 and 54 for SDV#1 experiment and days 16, 23, 30, 41 and 57 for SDV#2 experiment (Figure 1). Whole fish (SDV#1, mean weight of 0.9 g) or a piece of the skeletal muscle from the flank of each fish (SDV#2, mean weight of 3.3 g) were fixed in 10% neutral-buffered formalin for 7 days at 4 °C and embedded in paraffin. Five-micrometer sections were cut and stained with haematoxylin and eosin. To determine the distribution of the virus in the skeletal muscle tissue, immunohistochemistry analysis was performed using a primary mAb directed against E2 glycoprotein (17H23; [11]), diluted 1:2000 in sterile phosphate-buffered saline (PBS) and incubated overnight at 4 °C. Primary antibody was visualized with the Histofine Simple Stain MAX-PO kit (Histofine Biosciences inc); colour was developed with 3-Amino-9-EthylCarbazole (AEC chromogen; BD Pharmingen). The sections were then counterstained with Meyer’s haematoxylin. Immunofluorescence co-labelling was also performed on infected fish sections using the anti-E2 mAb diluted 1:2000 and a rabbit polyclonal antibody (pAb) against Pax7 (ab1; Sigma-Aldrich) diluted 1:500, as primary antibodies. Alexa Fluor 594- and 647-conjugated anti-mouse or -rabbit immunoglobulin secondary antibodies (Life Technologies) were used at 1:2000. Finally, fish sections were mounted in DAPI-ProLong Gold (Life Technologies) and visualized directly with a UV-light microscope (Carl Zeiss).

### Primary culture of rainbow trout satellite cells and SDV infection

Satellite cells were obtained from immature rainbow trout (between 5 and 10 g in weight) as previously described by Gabillard et al. [27]. Briefly, fish were euthanized and white epaxial muscle was excised and minced mechanically with a sterile scalpel in cold (4 °C) GMEM-HEPES supplemented with 15% of horse serum (Fisher Scientific) and containing penicillin (100 IU/mL), streptomycin (100 µg/mL) and amphotericin B (0.25 µg/mL). The tissue fragments were washed twice and an enzymatic digestion was performed with type IA collagenase (Sigma-Aldrich) at a final concentration of 0.1% for 1 h at 18 °C under gentle agitation and in the dark. After a low speed centrifugation (5 min at 300×*g*), the pellet was washed twice and resuspended (5 mL/g of muscle) in GMEM-HEPES containing 0.1% of trypsin (Sigma-Aldrich) and in absence of serum. After 20 min at 18 °C, the supernatant was harvested after centrifugation for 1 min at 300×* g* and diluted in 20 volumes of GMEM-HEPES containing serum to block trypsin activity. In parallel, the pellet underwent a second trypsin digestion and the supernatant after trypsin inactivation was pooled with that of the first digestion. The total supernatant containing the extracted cells was centrifuged 15 min at 300×*g* and the resulting pellet was resuspended and submitted to mechanical trituration through 5 mL pipettes. The cell suspension was successively passed through 100- and 40-µm nylon cell strainers. The cells were then centrifuged 15 min at 300 ×* g* and the cell pellet was carefully resuspended in GMEM-HEPES containing 10% of FBS and antibiotics. Finally, the cell number was determined using Malassez counting chamber, and then diluted to approximately 1.5 × 10^6^ cells/mL.

Culture plates were coated with poly-l-lysine (BD Biosciences) at a concentration of 8 µg/cm^2^ for 10 min at 18 °C and with laminin (BD Biosciences) at a concentration of 2 µg/cm^2^ overnight at 18 °C. The enrichment in satellite cells is based on the high affinity of these cells to laminin. Therefore, the crude cell suspension was seeded, allowed to bind to laminin for 30 min at 18 °C and finally washed to remove non adherent cells. Cell culture was performed with complete medium GMEM-HEPES supplemented with 10% of FBS and containing antibiotics at 18 °C. Cell monolayers in 12-well plates were washed 24 h later and infected with SDV at different multiplicity of infection (MOI). After 1 h of adsorption, the inoculum was removed, the cell monolayer was washed twice, and medium samples (0.2 mL of the 1-mL overlay) were taken (zero time point) and replaced by an equivalent volume of fresh medium supplemented or not with 2% of FBS. At different days post-infection (days 1, 3, 5, 7, 9, 11, 15, 18, 23, 30 and 73), supernatant aliquots were harvested, stored at −80 °C, and analysed later by plaque assays. At 73 days post-infection, cells monolayers were fixed and immunostained using anti-E2 mAb (see below).

### Indirect immunofluorescence assay

Rainbow trout satellite cells were passaged into 12-well plates (at a concentration of 2.5 × 10^5^ cells per well) 24 h prior to infection by SDV. At 3 days post-infection, cells were fixed with a mixture of ethanol and acetone [1:1 (v/v)] at −20 °C for 15 min. Antigen detection was performed by incubation with anti-E2 mAb and anti-desmin pAb (Dako) diluted 1:10 000 and 1:200, respectively, in PBS1x/0.05% Tween 20 for 45 min at room temperature. Cells were then washed three times, incubated with Alexa Fluor 488- and 594-conjugated anti-mouse or anti-rabbit immunoglobulins (Invitrogen), respectively, for 45 min at room temperature, washed again and mounted in Dapi Fluoromount G medium (Southern Biotech). Finally, cell monolayers were visualized directly with a UV-light microscope (Carl Zeiss).

## Results

### Experimental infection of juvenile rainbow trout by SDV: clinical signs, mortalities and virological examination

Two series of rainbow trout infection by SDV, namely SDV#1 and SDV#2, were performed using animals issued from the same in vitro fecundation but at two different mean weights, 0.9 and 3.3 g, respectively. These animal sizes were selected based on the fact that fish mortality rate decreases proportionally with fish age. Therefore, animals with a mean weight of 0.9 g were presumed highly sensitive to SDV and, in contrast, those with a mean weight of 3.3 g an intermediary sensitive stage allowing thus to keep enough surviving individuals. In both experiments, fifty juvenile rainbow trout were infected by immersion in a water bath containing 5 × 10^4^ PFU/mL of SDV, in duplicate. In the first tank, mortalities were recorded each day over a 2-month period of time as shown in Figure 1. Fish of the second tank were harvested at different time post-infection (see arrows on Figure 1) for histopathology and immunohistochemistry (IHC) analyses. Concerning SDV#1 experiment, mortality started at day 20 and cumulative percent of mortality was 72% at day 60 (Figure 1), as previously described for this animal size [4]. During the acute phase of the disease (days 20–35), the moribund fish displayed pathological signs including exophthalmia, pale gills, abdominal congestion, presence of ascites, darkening and congestion of the spleen, darkening of the head kidney and on few fish, haemorrhages could be observed on the fins and operculum and light petechiae on the abdomen. The virus was successfully recovered from ascitic fluids and a pool of organs (kidney, spleen, heart, and pancreas) from randomly euthanized animals harvested at day 26 post-infection. The viral titres were 1 × 10^5^ and 1.25 × 10^5^ PFU/mL, respectively. Concerning SDV#2 experiment performed with older individuals, the mortality was delayed compared to SDV#1 and started at day 26 post-infection. The cumulative percent of mortality was lower and reached only 52% at day 60 (Figure 1). The virus was successfully recovered from serum samples from randomly euthanized animals. The viral titres were very high and reached 2 × 10^10^ and 2 × 10^8^ PFU/mL, at day 16 and day 23 post-infection, respectively, just before the symptom appearance. No mortality was registered in the mock-infected trout control of each experiment during the same period of time.

### Virus-induced atrophy of the skeletal muscle and tissue regeneration in surviving trout

**Figure 2:**
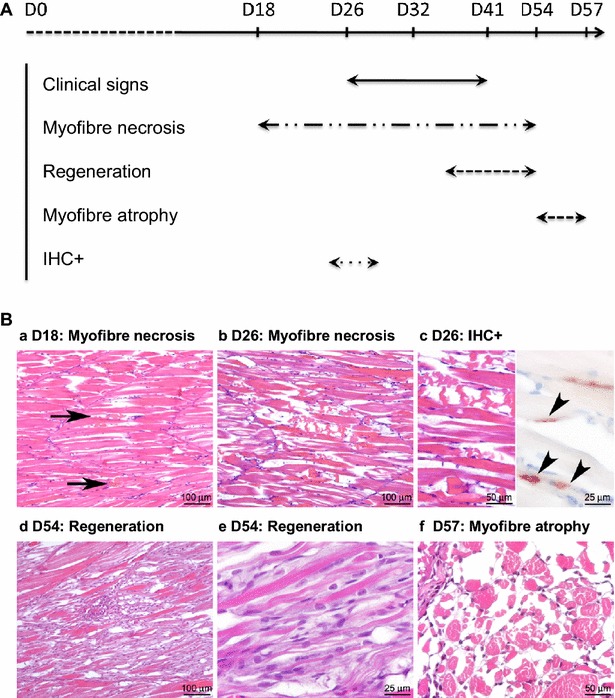
**Kinetics of sleeping disease lesion appearance in the skeletal muscle of experimentally infected rainbow trout through histopathology and immunohistochemistry studies.** **A** Time course of clinical and histological signs. **B** Histological and immunohistochemical characterisation of skeletal muscle pathology following SDV infection. *a* First lesions were observed 18 days post-infection. They were characterized by focal myofiber necrosis (arrows). *b* The severity of myofiber necrosis increased until day 26; *c* at this time point, a positive immunolabelling of SDV E2 antigens was detected at the periphery of necrotic myofibers (arrowheads). *d* At day 54 post-infection, a diffuse regeneration of the skeletal muscle was observed, with infiltration of macrophages and activation/differentiation of satellite cells, *e* that can fuse to form new myofibers, displaying aligned central nuclei. *f* Fifty-seven days post-infection, skeletal muscle was regenerated but a high proportion of myofibers was atrophied.

At different time point post-infection, 4 fish were randomly harvested from each experiment: days 13, 18, 26, 32, 39 and 54 for SDV#1 and days 16, 23, 30, 41 and 57 for SDV#2. Whole fish (SDV#1) or a piece of the skeletal muscle from the flank of each fish (SDV#2) were fixed and then prepared for histopathology and IHC analyses. Even if the infected fish did not display any clinical signs before day 26, the histopathological analysis revealed marked lesions characteristic of SDV infection in the exocrine pancreas together with an important presence of SDV antigens in this tissue at day 13 post-infection, as previously reported [11, 14], (data not shown). Concerning the skeletal muscle, lesions appeared 18 days post-infection (Figure 2A). These lesions were multifocal, more severe in the anterior part of the fish, and characterised by randomly distributed myofibers displaying segmental hypercontraction, loss of normal cytoplasmic striation, and cytoplasm fragmentation (necrotic myofibers) (Figure 2B). These affected cytoplasms also displayed a flocular or granular appearance, or could be fragmented. At day 26 post-infection, the density of necrotic myofibers increased and spread to the entire skeletal muscle system. Inflammation was minimal, only few inflammatory cells were indeed detected in the endomysium and no evidence of regeneration was observed until day 39 post-infection. The regeneration phase, from day 39 to 57 post-infection, was characterized by an infiltration of macrophages associated with a proliferation of myoblasts. At the end of this process, in the regenerated foci, a high density of small myofibers could be detected (myofiber atrophy) (Figure 2B).

**Figure 3:**
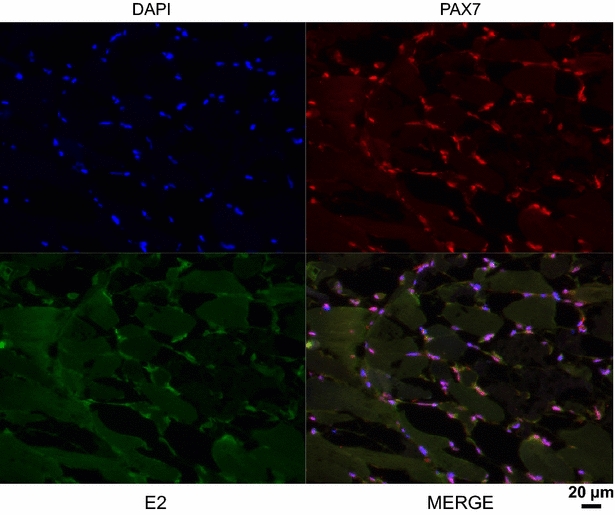
**Muscle satellite cells are cellular targets of SDV in rainbow trout.** Indirect immunofluorescence was performed on SDV-infected fish section 26 days post-infection. Satellite cells were stained using an anti-Pax7 pAb (red) and an anti-SDV E2 mAb (artificially coloured in green). The nuclei were stained with DAPI.

The detection of the SDV antigens was transient and IHC only revealed the presence of viral antigens 26 days post-infection (Figure 2B). The viral antigens (structural E2 glycoprotein) were detected in the necrotic foci, centred on what we suggested to be small peripheral or endomysial mononucleated cells. The cell type was further characterized by immunofluorescence co-labelling assay performed on infected fish sections using the anti-E2 mAb together with an anti-Pax7 pAb, a transcription factor specific to myogenic precursor cells in vertebrates [28]. Pax7 labelling was associated with small mononucleated cells located at the periphery of mature muscle fibers, reminiscent of satellite cells (Figure 3). Some Pax7-positive cells were also found positive for SDV antigens at 26 days post-infection, thus demonstrating that SDV exhibits a particular tropism for rainbow trout satellite cells.

### Ex vivo SDV infection model of rainbow trout satellite cells

**Figure 4:**
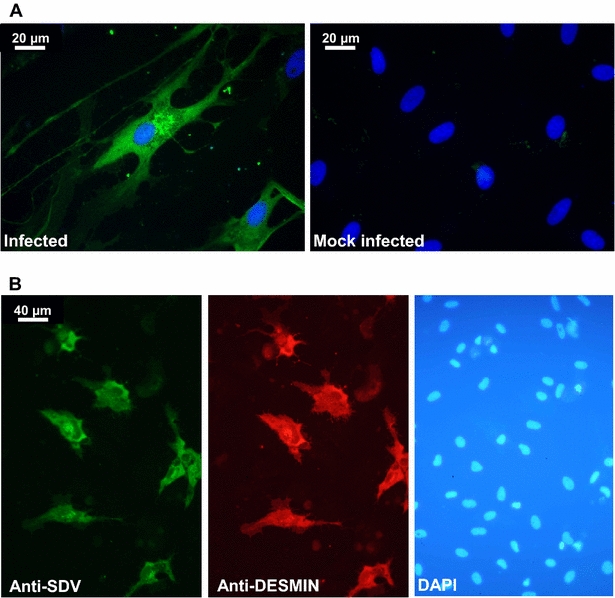
**Ex vivo SDV infection model of rainbow trout satellite cells.** Primary culture of rainbow trout satellite cells were infected or not with SDV at an MOI of 10. At 3 days post-infection, cells were immunolabelled with the anti-E2 mAb (**A**) or with the anti-E2 mAb together with an anti-desmin pAb (**B**). The nuclei were stained with DAPI.

**Figure 5:**
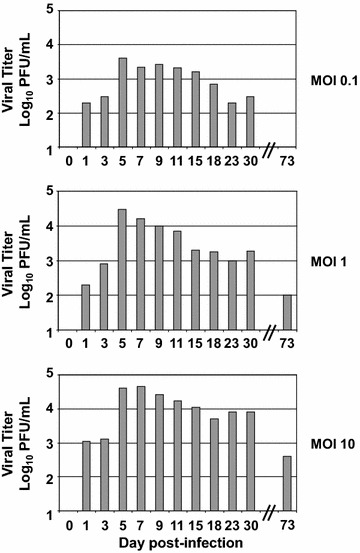
**Growth kinetics of SDV in rainbow trout satellite cells.** Primary culture of rainbow trout satellite cells were infected with SDV at an MOI of 0.1, 1 and 10 PFU per cell. Supernatant aliquots were taken at the indicated day post-infection and viral titres were determined later by indirect immunofluorescence using the anti-E2 mAb. The lower limit of detection for virus was 2 log_10_ PFU/mL. Each virus titration was done in duplicate. Means are shown. The standard errors were calculated, but the bars are not shown because the errors were very small.

**Figure 6:**
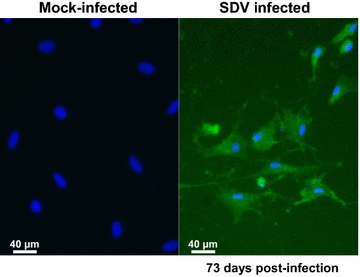
**Persistent SDV antigen detection in primary culture of rainbow trout satellite cells at 73 days post-infection.** Primary culture of rainbow trout satellite cells were infected or not with SDV at an MOI of 10. At 73 days post-infection, cells were fixed and immunolabelled with the anti-E2 mAb. The nuclei were stained with DAPI.

To confirm that particular tropism of SDV for rainbow trout satellite cells, we performed an in vitro approach. Primary cell cultures of rainbow trout satellite cells were tested for their sensitivity to SDV infection. Thus, undifferentiated muscle satellite cells were inoculated with SDV at a multiplicity of infection (MOI) of 10. At 3 days post-infection, viral antigens could be detected in the cytoplasm of the infected cells by indirect immunofluorescence using a mAb directed against the structural E2 glycoprotein (Figure 4A) [11]. No signal could be detected on mock-infected cell cultures. Moreover, SDV-infected cells were also immunolabelled with a pAb raised against the desmin, another specific myogenic marker, demonstrating that rainbow trout satellite cells are targeted by SDV (Figure 4B). Interestingly, the other cell types, which do not express the desmin marker and found in the primary culture of satellite cells (see DAPI-labelling in Figure 4B), were not found immunoreactive to SDV E2 antibodies. Regardless of the MOI used, a cytopathic effect (CPE) could be observed in SDV-infected cell cultures between 5 and 10 days post-infection. The CPE was characterized by scattered foci of rounded cells and seemed to be stronger when the infections were performed in absence of serum (data not shown). Analysis of viral yields by plaque assay revealed that a productive SDV replication occurred in rainbow trout satellite cells. The viral yield reached a value of 4 × 10^3^ to 4.5 × 10^4^ PFU/mL, depending on the MOI used, between 5 and 7 days post-infection (Figure 5). The viral yield was maintained throughout at least a 30-day period with a limited variation of approximately tenfold and the CPE was never very extensive. Surprisingly, infected cells could be kept at least 73 days post-infection and viral yield was still detected in the supernatant. Moreover, these cells were still positive for SDV E2 antigen as shown in Figure 6 and displayed highly condensed nuclei compared to mock-infected cells.

## Discussion

In the present work, we described a new and valuable model to study alphavirus-induced pathogenesis and tropism in the skeletal muscle. This relevant model which reproduces the full pathogenesis as observed in the field is based on the infection of juvenile rainbow trout by a salmonid alphavirus which does not require any injection since the virus is naturally transmitted via a water-borne route [10, 29]. This natural infection allowed following and describing in details the kinetics of development of musculoskeletal lesions induced by SDV, the severe myonecrosis and atrophy of the myofibers, and the delayed muscle regeneration in survivors. These data confirmed previous detection of viral RNA and antigens in skeletal muscle of infected animals [11, 30–32] and constitute the first observation of a specific SDV tropism for the muscle satellite cells in vivo. E2 glycoprotein from SDV was detected in Pax7-positive cells located at the periphery of mature muscle fibers of infected fish demonstrating that SDV infects and replicates in trout muscle satellite cells. In addition to anatomic location, satellite cells are characterized by specific markers such as the paired box transcription factor Pax7. The transcription factor Pax7, together with Pax3, plays essential roles in the early specification, migration and myogenic differentiation of satellite cells [33, 34]. Furthermore, it was demonstrated that Pax-7 expressing cells are absolutely required in fiber regeneration in adult mammalian muscle thus providing evidence that Pax7-positive cells are muscle stem cells [35, 36]. Zebrafish, like other teleost species and amphibians, have also muscle progenitor cells that share similar characteristics to those of mammalian. They express the key marker Pax7 and participate in muscle repair [28, 37].

In parallel, we developed an in vitro model of rainbow trout satellite cell infection, showing that SDV could efficiently infect primary cell cultures of satellite cells although virus production was somewhat moderate. To our knowledge, this constitutes the first model of long-lasting infection of primary satellite cells by an alphavirus. This specific tropism for myogenic cells has already been documented for CHIKV both in vitro and ex vivo [22]. CHIKV antigens were detected in skeletal muscle satellite cells of infected human biopsies, during both the acute phase of CHIKV infection and the late recurrent symptomatic phase of the disease, with muscle necrosis and an inflammatory infiltrate observed in late phase. Such a tropism during naturally acquired infection in human, could also be observed during experimental infection of a murine model [23], as well as in human primary cultures of satellite cells [22]. Thus, the SDV-infected trout model could be an alternative to study alphavirus-induced myopathy in a more accurate context compared to some mouse models presenting immune deficiency or immunological immaturity (for review see [38]).

The viral-induced cytopathic effect of these particular muscle cells might have important consequences on the skeletal muscle physiology that could explain the slow recovery and reduced muscle growth observed in survivors [13, 32]. Indeed, the loss of exocrine pancreatic tissue resulting in inappetence and defective uptake of nutrients [32, 39] together with the loss of muscle progenitor cells slowing down the muscle regeneration might be the reason of the low growth rate observed in surviving fish. In addition, previous results mentioned that clearance of the virus did not occur in all challenged fish which might lead to persistent chronic inflammation and thus delaying even more the full recovery in surviving fish [30, 31, 40]. These observations are supported by the long lasting in vitro replication of SDV in trout skeletal muscle progenitors. Indeed, SDV protein expression and infectious virus production although very low could be detected up to 2 months post-infection. Some alphaviruses infecting mammals, such as CHIKV, Sindbis virus (SINV) and Ross River virus (RRV), can also persist in specific tissues or cell populations despite a strong immune response and an apparent clearance of virus from the circulation [41–44]. All together these data reinforce that viral replication in muscle cells is closely associated with acute and chronic myalgia and suggest in the case of SDV that carrier state in salmonids is possible, as previously mentioned by several authors [40, 45].

In a recent study, Heidari et al. analysed the gene expression in skeletal muscle in response to SAV3 infection in Atlantic salmon (*Salmo salar*) [32]. They focused on a few selective genes involved in the antiviral immunity and related to the metabolic processes. They observed that the expression of key genes involved in the innate and adaptive immune responses was significantly up-regulated and occurred concomitantly with the peak of viral replication in the muscle tissue. Moreover, *atrogin*-*1* and *MurF1* (muscle-specific RING finger 1) gene expression was also induced. Atrogin-1 and MurF1 are key ubiquitin E3 ligases that are important regulators of ubiquitin-mediated protein degradation in skeletal muscle. In mammalians, all conditions of muscle atrophy studied so far have shown induction of these proteins which lead to increased protein degradation through the ubiquitin–proteasome system [46]. Interestingly, *atrogin*-*1* gene expression was highly increased in parallel to the interferon response. However, it is unclear whether both responses are directly linked to the viral infection of the muscle tissue or independently induced. Indeed, it has been shown that the *atrogin*-*1* gene was efficiently induced by an inflammation stimulus independently to any pathogen infection in Atlantic salmon [47]. Thus, it could be of interest to analyse whether the productively infection of trout muscle satellite cells by SDV regulates activation of muscle cell differentiation and simultaneously induces similar changes in expression of genes encoding antiviral actors and/or muscle atrophy mediators.
